# Tracking Cholera through Surveillance of Oral Rehydration Solution Sales at Pharmacies: Insights from Urban Bangladesh

**DOI:** 10.1371/journal.pntd.0004230

**Published:** 2015-12-07

**Authors:** Andrew S. Azman, Justin Lessler, Syed Moinuddin Satter, Michael V. Mckay, Azharul Khan, Dilruba Ahmed, Emily S. Gurley

**Affiliations:** 1 Johns Hopkins Bloomberg School of Public Health, Baltimore, Maryland, United States of America; 2 International Centre for Diarrhoeal Disease Research, Bangladesh, Dhaka, Bangladesh; 3 RTI International, Nairobi, Kenya; Santa Fe Institute, UNITED STATES

## Abstract

**Background:**

In Bangladesh, pharmacy-purchased oral rehydration solution (ORS) is often used to treat diarrhea, including cholera. Over-the-counter sales have been used for epidemiologic surveillance in the past, but rarely, if ever, in low-income countries. With few early indicators for cholera outbreaks in endemic areas, diarrhea-related product sales may serve as a useful surveillance tool.

**Methodology/Principal Findings:**

We tracked daily ORS sales at 50 pharmacies and drug-sellers in an urban Bangladesh community of 129,000 for 6-months while simultaneously conducting surveillance for diarrhea hospitalizations among residents. We developed a mobile phone based system to track the sales of ORS and deployed it in parallel with a paper-based system. Our objectives were to determine if the mobile phone system was practical and acceptable to pharmacists and drug sellers, whether data were reported accurately compared to a paper-based system, and whether ORS sales were associated with future incidence of cholera hospitalizations within the community. We recorded 47,215 customers purchasing ORS, and 315 hospitalized diarrhea cases, 22% of which had culture-confirmed cholera. ORS sales and diarrhea incidence were independently associated with the mean daily temperature; therefore both unadjusted and adjusted models were explored. Through unadjusted cross-correlation statistics and generalized linear models, we found increases in ORS sales were significantly associated with increases in hospitalized diarrhea cases up to 9-days later and hospitalized cholera cases up to one day later. After adjusting for mean daily temperature, ORS was significantly associated with hospitalized diarrhea two days later and hospitalized cholera one day later.

**Conclusions/Significance:**

Pharmacy sales data may serve as a feasible and useful surveillance tool. Given the relatively short lagged correlation between ORS sales and diarrhea, rapid and accurate sales data are key. More work is needed in creating actionable algorithms that make use of this data and in understanding the generalizability of our findings to other settings.

## Author Summary

In Bangladesh, people often purchase oral rehydration solution (ORS) at their neighborhood pharmacy to treat diarrhea, including cholera. Over-the-counter sales have been used for epidemiologic surveillance, but rarely in low-income countries. With few early indicators for cholera outbreaks in endemic areas, diarrhea-related product sales may be a useful surveillance tool. We tracked daily ORS sales at pharmacies and drug-sellers in an urban Bangladesh community with both a mobile phone and paper-based system while conducting surveillance for diarrhea hospitalizations among residents. We found that increases in ORS sales were significantly associated increases in hospitalized diarrhea cases up to two days later and hospitalized cholera cases up to one day later. Our findings suggest that surveillance systems based on over-the-counter product sales may be a feasible and useful way to detect outbreaks in low-income settings and that mobile technology may make it even easier to collect implement.

## Introduction

Despite well over a hundred years of research on the disease, cholera remains a threat to public health in many parts of the world, causing more than 2–3 million cases each year and over 100,000 deaths [1]. While many of the global cholera deaths come from large unexpected outbreaks, thousands die in highly endemic settings, like Bangladesh, each year [2]. Any improvements to cholera surveillance systems that can provide an early warning of an increase in transmission may provide an opportunity to intervene with vaccine or water and sanitation improvements, averting cases and saving lives.

Most cholera surveillance systems in endemic countries are passive and clinic-based; ultimately capturing only the severe cases that are able to access healthcare services. Active case-identification systems at the community level are rare due to the costs involved; however, opportunities may exist in some areas to tailor novel custom surveillance systems to the ways in which people seek care for diarrhea. Pharmacies often serve as the first point of contact with the healthcare sector when individuals become ill. In Bangladesh, people commonly use oral rehydration solution (ORS) purchased at pharmacies to treat diarrhea, including *Vibrio cholerae*, in part due to years of mass educational campaigns and social marketing [3–6]. Trends in the sales of different diarrhea-related products may serve as a useful tool for detecting increases in cholera within a community, perhaps before clinic-based surveillance systems detect the signal of an outbreak.

Pharmacy sales have been used in surveillance within high-income countries to gain insight into disease trends including diarrhea [7–10] and influenza [11]. To our knowledge, this tool has not been employed in low resource settings, where disease surveillance is often much poorer and pharmacy sales are usually not routinely tracked electronically. Surveillance systems to track pharmacy sales may provide even greater utility in countries like Bangladesh than in high-income countries, given the lower baseline quality of disease surveillance systems within the country.

We implemented real time mobile phone- and paper-based surveillance systems for ORS sales at pharmacies in an endemic community in Dhaka, Bangladesh and compared it with active, case-based diarrhea surveillance at local hospitals. Our objectives were to determine if the mobile phone system was practical and acceptable to pharmacists and drug sellers, whether data were reported accurately compared to a traditional paper-based system, and whether ORS sales were associated with future incidence of cholera hospitalizations within the community.

## Methods

### Study Site

Arichpur, is a 1.2 km^2^ urban community located 15 km north of Dhaka, Bangladesh in the Tongi sub-district. Arichpur has approximately 129,000 residents living in 29,000 households where many nuclear families share one room and up to 10–15 families may share a stove, toilet, and water source. Although the cholera burden in Arichpur has not been estimated, patients from this area frequently visit the icddr,b (International Centre for Diarrheal Disease Research) cholera treatment center in Dhaka and have suffered from outbreaks of other water-borne diseases including hepatitis E [12].

### ORS Sales Surveillance

**Fig 1:**
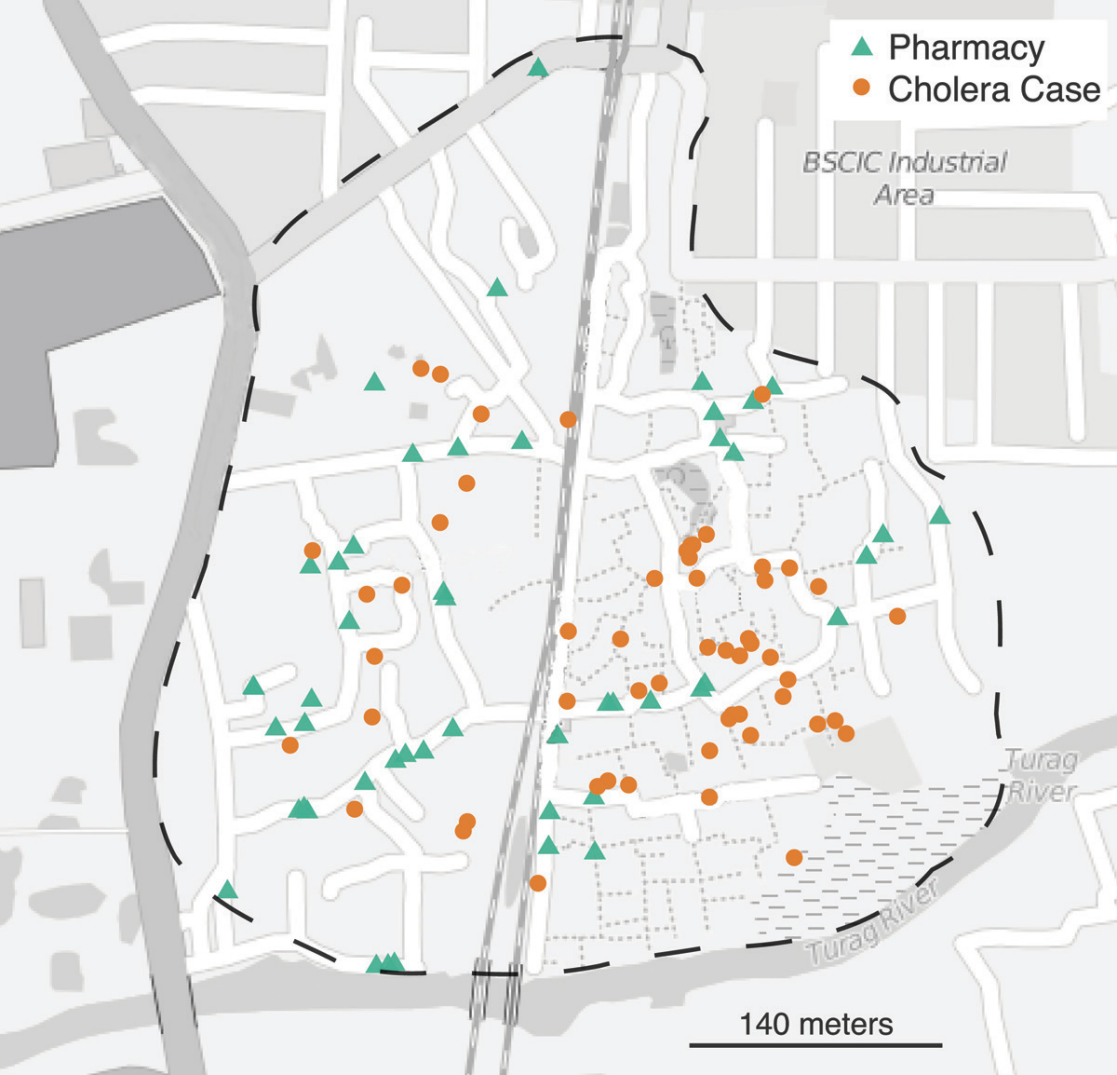
Locations of enrolled pharmacies and confirmed cholera cases. Dashed line represents approximate boundaries of study area (Arichpur), green triangles represent enrolled pharmacies where ORS sales were tracked and orange circles represent the locations of confirmed cholera cases (n = 53 enrolled in household visit portion of the study). Base map courtesy of www.openstreetmap.org.

All pharmacies and drug sellers in Arichpur were enumerated and mapped. We then enrolled 50 pharmacies, out of 124 due to cost and human resource constraints, with equal probability, replacing those who declined to participate with the next on the list (Fig 1). We used uniform random selection, as opposed to a more advanced surveillance site selection [13,14] technique, as no prior sales data, nor high-resolution population data, were available. At each pharmacy we identified the employees primarily responsible for diarrhea-related sales and trained each on the use of paper forms and phone reporting systems. All pharmacies were enrolled between 25 March and 30 April 30, 2013, and continued reporting to the surveillance system through October 2013.

Pharmacy employees reported ORS sales through two parallel systems; a paper based system, and a phone-based interactive voice response system [15]. With the paper-based system, employees recorded the number of customers each day and the number of ORS packets purchased by each. The paper forms were collected by study staff at the end of each month and were entered into an electronic database at icddr,b.

To initiate the phone-based reporting system, we first collected mobile phone numbers of the key sales staff at each pharmacy and entered them into the phone system database. We instructed pharmacy staff to call the system number and hang-up after the first ring (this allowed the pharmacy personnel to avoid incurring the cost of the call). This ‘missed call’ triggered the phone system to (1) associate the caller with a specific pharmacy, and (2) call the individual back with a prerecorded message (in Bengali) asking ‘how many ORS customers have you had today?’ In response, the sales person entered the digits corresponding with the number of customers. The system then read back the number they entered and provided and an opportunity to correct any mistakes. Following the call, the system sent a text message back to the sales person confirming that their report had been received. If no calls were received from a pharmacy within a 36-hour period, the system automatically called each number associated with the pharmacy a maximum of two times to attempt to collect data on sales. If none of these attempts led to a successful sales report, an SMS alert was automatically sent to the field team and a phone call or in-person visit was made by study staff to troubleshoot and to collect data from the missing period.

Field teams visited each pharmacy weekly to collect the paper-based forms and solve any difficulties faced by the pharmacy staff in completing the forms or using the phone-based system. Pharmacies were paid approximately $5 USD (a loaf of bread costs approximately $0.50 USD at the time of writing this manuscript) per month as compensation for their participation.

### Cholera Surveillance

Our formative research indicated that the majority of individuals from Arichpur who seek care at hospitals for diarrhea attend one of two main hospitals, the Tongi Sub-district Hospital and the icddr,b Dhaka Hospital. From April through October 2013, we screened patients at both hospitals on admission to identify Arichpur residents aged 2-years or older hospitalized with diarrhea. Consenting patients provided a stool sample for cholera culture. Study staff visited households of confirmed cholera cases within 3 days of the hospital visit and all household members with diarrhea older than 2 years were asked to provide a stool sample for cholera testing.

Stool samples from households and the Tongi hospital were transported in Cary-Blair media to the microbiology lab at the icddr,b Dhaka hospital. All samples were cultured using standard methods onto taurocholate-tellurite-gelatin agar media [16]. In addition, a portion of each sample was enriched in Bile Peptone broth overnight and then cultured for enrichment to improve specificity [17].

### Ethical Approval

Trained study staff asked each eligible patient or their guardian for their written informed consent to participate in the study. This study was approved by the Johns Hopkins University Institutional Review Board and the icddr,b Ethical Review Committee.

### Data Analyses

#### Comparison of mobile phone and paper-based surveillance

We compared the number of ORS customers reported through the mobile phone- and paper-based systems using Pearson’s correlation coefficient. We also compared the average estimated number of customers per day per pharmacy between the two methods. We estimated the concordance of periods with higher than average sales based on both phone-based and paper-based data using Cohen’s Kappa statistic [18]. We assumed that the paper-based system was more accurate based on discussions with pharmacy staff and exploratory analyses; therefore our main analyses rely on the paper-based system data except when comparing the two systems.

#### Trends in ORS sales, cholera, and non-cholera diarrhea

To distinguish periods of higher than expected incidence, we modeled diarrhea incidence (cholera-specific and all diarrhea) with a quasi-Poisson regression model and considered days where the number of cases (presenting to the hospital) exceeded the 95% confidence intervals as days of higher than expected incidence. Since we likely missed cases from the community, we explored both smoothed (3-day moving average and splines, [19]) and crude (i.e., unsmoothed) versions of the community-wide epidemic curves in analyses. All results presented are from analyses with the raw data unless otherwise noted. We estimated the presence of space-time clusters of confirmed cholera cases using the Space-Time Permutation Model implemented in SatScan [20] and only consider those with a permutation p-value less than 0.05 as significant.

To understand variations in sales within individual pharmacies over time, we normalized pharmacy-specific sales (i.e., the number of customers purchasing ORS each day) by subtracting the mean and dividing by the standard deviation of the number of daily customers from each pharmacy. Values greater than zero represent larger than average sales and those less than zero represent less than average sales. We used the mean of these normalized pharmacy specific sales as a measure of the average excess sales within the community. We refer to these as ‘community-wide excess sales’ throughout the paper. We used a multi-dimensional clustering algorithm to reduce the sales trajectory of each pharmacy to a single dimension (similar using the first principal component in principal component analysis) in order to group pharmacies together by sales trajectory and to explore possible relationships in sales trends between pharmacies [21]. To understand how sales tended to cluster spatially, we modeled the relationship between the geographic distance between pharmacies and their scaled sales trajectory with generalized additive and linear spline models.

### Association between Community-wide Excess Sales and Diarrhea Hospitalizations

We first explored the time-lagged relationship between community-wide excess ORS sales and hospitalized diarrhea cases (separately for cholera-confirmed and all diarrhea) by estimating the cross correlation function at different time lags (+/- 14 days) along with asymptotic 95% confidence intervals. We then modeled a series of lagged relationships with generalized linear models assuming a Poisson or quasi-Poisson error distribution adjusting for one potential confounder, the mean daily temperature. We used the Akaike Information Criteria (AIC) [22] from Poisson models at different lags to choose the best lag for each outcome (S1 and S2 Tables). Due to the over-dispersed nature of the diarrhea outcomes, and based on previous publications [23,24], we used quasi-Poisson models for the main analyses.

All analyses were conducted using the R statistical programming language (version 3.0.3) and many plots were produced using the ggplot2 package [25,26]. Source code and data for these analyses are available at https://github.com/HopkinsIDD/cholera-ORS/.

## Results

### Hospitalized Diarrhea Cases

**Fig 2:**
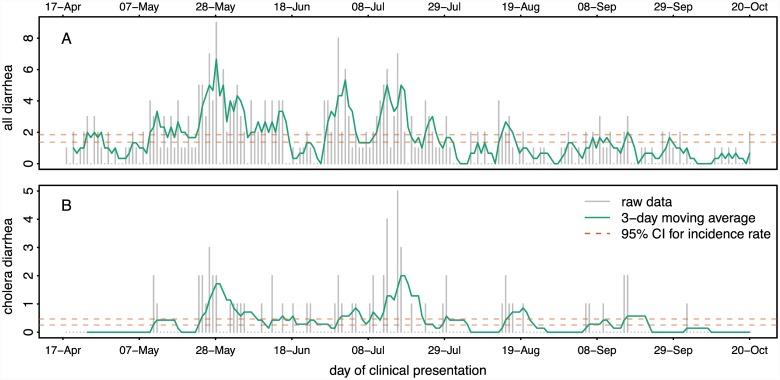
All diarrhea (A) confirmed cholera diarrhea (B) epidemic curves. Grey bars illustrate the number of cases each day from both surveillance sites combined, with the green line showing their 3-day moving averages. The dashed orange lines represent the 95% Poisson confidence intervals. Periods of significantly elevated transmission are noted with an orange dash below the x-axis.

We identified 315 residents of Arichpur hospitalized for diarrhea from 17 April to 30 October 2013 (Fig 2A); 155 from the icddr,b Dhaka Hospital and 160 from Tongi District Hospital. Among these, 69 (22%) had a positive stool culture for cholera with 28 (41%) of these admitted to the Tongi Hospital (Fig 2B). While we did not collect age data for all diarrhea cases, the median age of the 53 confirmed cholera cases for which we have age-data on was 22 (IQR 14–35). These confirmed cholera cases were well dispersed throughout the study area though we detected two significant space-time clusters of cholera, one from 3-July-2013 to 8-July-2013 (Cluster 1) and the other from 20-August-2013 to 29-September-2013 (Cluster 2, S1A Fig).

On 31% of days (62/197) no eligible patients were hospitalized for diarrhea. We detected confirmed cholera on 23% (45/197) of the study days. On days we identified diarrhea patients, we enrolled between 1 and 9 cases per day, up to 5 of which had cholera cultured from their stool (Fig 2).

### ORS Sales

We initially enrolled 50 out of the 124 enumerated pharmacies into our surveillance network and randomly substituted two new pharmacies for two that dropped out within the first month of the study. Few pharmacies had days with no sales report after their initial enrollment, with an average of 0.28 days of missing data per pharmacy (range 0–2). In total we recorded 47,215 customers purchasing 140,614 packets of ORS over the study period. Pharmacies had an average of 5 ORS customers per day (range 0–68, interquartile range [IQR] 2–6) each purchasing on average 2.8 ORS packets (range 1–60, IQR 1.8–3.3). On average 37% percent of the ORS sales were intended for individuals less than 15 years old.

**Fig 3:**
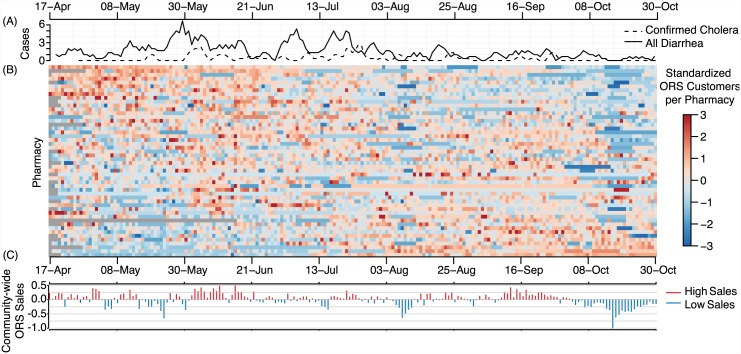
Smoothed epidemic curves for all diarrhea and confirmed cholera hospitalizations (A), standardized ORS customers by pharmacy (B) and mean community-wide excess ORS sales (C). In Panel B, each row in the colored matrix represents the standardized sales trajectory for a single pharmacy with colored pixels representing sales for each day. Red pixels represent days with higher than expected sales and blue pixels represent days with lower than expected sales (grey cells represent days with missing days). *Community-wide excess ORS sales* (C) for each day is the average of these standardized values across all pharmacies.

We found highly variable patterns in normalized ORS sales between pharmacies throughout the study period with no apparent synchrony in periods of higher or lower than expected sales (Fig 3B). For example, early on in the surveillance period a number of pharmacies had a period of higher than expected sales (e.g., pharmacies on the top rows of Fig 3B), while others had lower than expected sales (e.g., pharmacies on the bottom rows of Fig 3B). Community-wide excess sales, a statistic capturing deviations in ORS customers throughout the community, ranged from -1.0 in October to 0.5 in June (Fig 3C).

While we identified groups of pharmacies with similar ORS sale trajectories, we found no clear evidence that these clustered together geographically within the community. However, we found that pharmacies tended to have increasing similarity up to distances of about 100 meters from one another (S2 Fig), though this trend was not statistically significant (by *t*-test on linear spline regression model). Given that we found some evidence of space-time clustering of cholera cases, we explored whether pharmacy sales within the cluster were elevated compared to those outside of the cluster. We found that ORS sales during the clustering period from pharmacies inside the primary space-time cholera cluster tended to be higher than those outside the cluster (S1B Fig) though the differences were small.

#### Reliability, Feasibility, and Acceptability of Phone-Based Reporting System

The mobile phone-based system was used regularly (total of 8,862 phone reports throughout the surveillance period) by all but two of the initial pharmacies enrolled, and we found no evidence of a decreasing trend in reporting frequency suggestive of reporting fatigue, as the study progressed (S3 Fig). While study staff reported no major problems in use of the phone system, some of the challenges encountered included; (1) most pharmacies had no fixed telephone line so we were reliant on calling personal phones of staff, who change on a regular basis and (2) misunderstandings related to how to submit a report after a reporting delay. Finally, we experienced a few short periods where the phone system was down for maintenance and some of the pharmacies that tried unsuccessfully to report during the outage failed to report again until reminded by study staff within the proceeding few days.

Sales data from pharmacies reported through the phone-based surveillance system were moderately correlated with sales from the paper-based recording system and varied by pharmacy. The median correlation of daily sales between the two systems was 0.28 (IQR 0.17–0.47) with a maximum correlation of 0.72. Aggregation to weekly sales improves the median within-pharmacy correlation between the two reporting systems to 0.59 (IQR 0.41–0.74). The median weekly correlation between systems was 0.76 (IQR 0.67–0.85), which equates to 52% of the variance explained. While the exact numbers from each reporting system may have differed each day, we found that the two systems agreed 88% (Cohen’s Kappa = 0.52) of the days on whether sales were above or below average.

We also compared the cumulative sales between the two methods (S4 Fig). We found high variability between pharmacies with some pharmacies having close to identical cumulative sales curves, and some, who reported on the phone-based system less frequently, had quite different distributions. In general the phone-based reporting system cumulative sales reports tended to be less than those derived from the paper-based system with some notable exceptions (Pharmacies 264, 289 and 290 in S4 Fig).

### ORS Sales and Hospitalized Diarrhea

**Fig 4:**
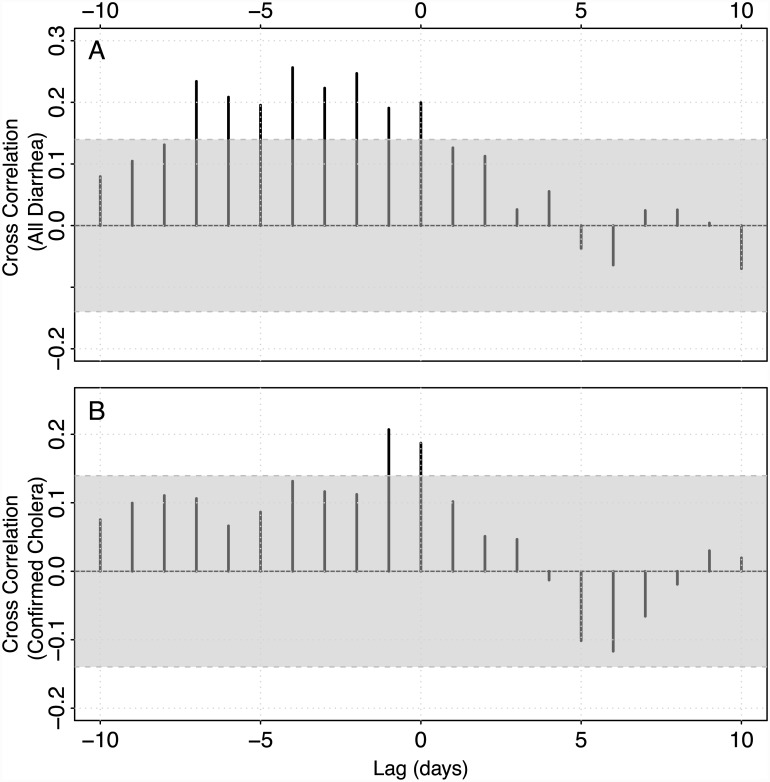
Cross correlation between mean community-wide excess ORS sales and all diarrhea (A) and cholera diarrhea (B). Each bar represents the value of the cross correlation function for a specific lag between ORS sales and a specific outcome. The lag values represent the number of days that sales come after the case, so negative values refer to situations where ORS sales precede diarrhea cases. The 95% confidence band is shown in grey.

In analyses unadjusted for mean daily temperature, ORS sales were significantly associated with incident hospitalizations for diarrhea up to 9 days later, with the peak association occurring 7 days after the sales. One additional community-wide excess ORS sale was associated with a 3.5 fold rise in diarrhea hospitalization risk seven days later (95% Confidence Interval (CI) 1.74–7.18). Similarly, using the cross correlation function, we found that diarrhea cases were significantly associated with community-wide ORS sales up to a week before (Fig 4A). While unadjusted analyses may be helpful for surveillance, daily temperature may confound our estimates of the relationship between ORS sales and diarrhea. In analyses adjusted for the mean daily temperature, the association between ORS sales and incident diarrhea was attenuated, with no significant association (Relative Risk [RR] 2.03, 95% CI 0.89–4.76) between ORS and diarrhea found in the best fitting (7-day lag) model. Mean daily temperature explained more of the variation in diarrhea incidence in this model, with a one degree Celsius increase in temperature (above the period mean) associated with a 14% (RR 1.14, 95% CI 1.02–1.28) increase in the risk diarrhea hospitalization risk seven days later. However, ORS sales were significantly associated with incident diarrhea hospitalizations up to 2-days later in adjusted models, with one additional community-wide excess ORS sale was associated with a 2.7 fold rise in diarrhea hospitalization risk two days later (95% CI 1.15–6.47). At short time lags (1–2 days), mean daily temperature was not independently associated with diarrhea incidence after adjusting for ORS sales.

ORS sales were associated with cholera-confirmed diarrhea hospitalizations only up to one day later in both unadjusted and adjusted models. The best fitting model and the cross correlation analysis suggested that the peak association occurred one day after the sale (Fig 4B). We find that one additional community-wide excess ORS sales unit was associated with an 11.1 fold higher cholera risk one day later (95% CI 2.46–53.44) in an unadjusted model and a 6.5 (95% CI 1.15–40.49) fold increase in a model adjusted for mean daily temperature. Mean daily temperature was not significantly associated with cholera hospitalizations in this best fitting model.

## Discussion

Through tracking the sales of ORS at pharmacies and conducting surveillance for hospitalized diarrhea cases in an urban Bangladeshi community, we find that ORS sales were significantly associated with hospitalized diarrhea cases up to nine days later in unadjusted models and two days later in adjusted models, and hospitalized cholera cases up to one day later. These results suggest that first-line healthcare providers could serve as the basis for a new avenue for disease surveillance in low-resource settings where traditional surveillance systems may not adequately capture disease trends in the community.

To our knowledge, this is the first study in a low resource setting to explore the association of over the counter sales with diarrhea incidence. Previous studies in North America and Europe, have found conflicting results on the utility of this approach for detecting outbreaks of GI illnesses [8,9]. One study in Canada, found over-the-counter sales of anti-diarrheals and anti-nauseants to increase contemporaneously with diarrheal cases (hospitalized and identified through case investigations) in outbreaks of cryptosporidium, campylobacter, and *E*. *coli* O157:H7 [9]. Another study, in California (USA), found no significant association of over-the-counter pharmacy sales and county-level reports of GI illness [8]. Local diarrheal disease epidemiology, care-seeking behaviors and statistical methods used to assess the relationships between sales and diarrhea incidence may be responsible for these apparent differences.

The estimated short time lag between ORS sales and cholera compared to that of non-cholera diarrhea could be due to differences in the natural histories of the putative pathogens. While the incubation period of cholera is unlikely to be significantly faster than many other common gastro-intestinal illnesses, our findings could be due to the relative rapidity of severe diarrhea caused by cholera [27, 28]. Alternatively, differences in care-seeking behavior in response to cholera and non-cholera diarrhea could contribute to differences in their lagged association with ORS sales.

While the phone-derived sales data reflected the data collected through the paper system, more work is needed to ensure that pharmacies regularly report and that gaps in reporting can be accurately and efficiently backfilled. With paper-based forms, this was often done through a review of the pharmacy records, but the phone-based system was only designed to ask whether the first report after a reporting gap was cumulative (i.e., did the current report include all the sales since the last report) or whether it pertained only to new sales that day. Future implementation of this system should improve on the ability to fill in non-reporting gaps. More research is needed on the ways in which people most easily interact with a phone-based reporting system (e.g., possibly the inclusion of SMS/text message-based reporting) to improve data quality. These modifications may ultimately lead to a viable electronic system producing timely data in low resource settings with similar data quality to paper forms.

Though promising, our findings come with a number of limitations. Our analyses are based on data from one small community over the course of a single, six-month, cholera season, limiting the generalizability of our findings. For these results to be more practically useful in outbreak detection, longer-term surveillance of both sales and disease is needed. With more data across seasons, a robust predictive model capable of continuously integrating new data points could be constructed, which could allow for diarrhea and cholera incidence forecasts or development of simple outbreak alert algorithms similar to those proposed for other diseases like influenza [29]. With an enhanced phone-based system and improved algorithms using data from a larger network of pharmacies, public health practitioners could receive actionable data on a daily basis, if not in real-time. This delay would likely be small enough to take advantage of the estimated lagged relationships between ORS sales and diarrhea (and cholera, 1–9 days) and improve the public health utility of this method.

Further exploration of the spatio-temporal patterns of ORS sales and their specificity for diarrhea and/or cholera cases is warranted. If sales in pharmacies around clusters of cases are elevated before cases appear at clinics, a modified version of our approach may not only provide a case alert about the influx of cases, but also the potential locations. Additionally, we illustrate the potential, yet not statistically significant, correlation in sales ORS trajectories for pharmacies within 100 meters of one another. With more data and alternative analyses, understanding this relationship between sales at different pharmacies could help in more efficiently choosing surveillance sites. We reduced each pharmacy’s sales trajectory to a single dimension with a clustering algorithm and this does fully capture the essence of the complex sales patterns. Future analyses could use additional dimension reduction techniques to characterize pharmacies’ sales patterns.

Moderate amounts of missing data, like what we observed with the phone-based system, make summary statistics like community-wide excess sales less useful due to the high variation in baseline sales between pharmacies. This measure is robust to randomly missing pharmacy reports but could lead to spurious results when missing data (i.e., non-reports) are associated with individual pharmacy sales and diarrhea in the community. If the relationship between ORS and diarrhea in the community is to be explored with more missing reports or missing reports that are thought to be non-random, community-wide sales should be modeled in a more structured manner.

Pharmacies and drug sellers in Dhaka sell an array of diarrhea-related products, including antibiotics; and many administer intravenous rehydration therapies. More research is needed to understand if different diarrhea-related products may better predict diarrhea and cholera incidence in the community, and whether they may do so with longer lead times. When conducting the formative research for this study we learned that ORS is often used for rehydration even in the absence of diarrhea on hot days. Unsurprisingly, we found that ORS sales within this study were significantly associated with mean daily temperature (S4 Fig). We also found that mean daily temperature was associated with incident diarrhea though not with cholera. Previous analyses of non-cholera diarrhea from Bangladesh have shown a similar trend of increasing diarrhea with increasing temperature [30,31], though a more nuanced relationship with cholera and temperature has been noted in other analyses [32]. Our estimates of the relationship between lagged ORS sales and diarrhea and cholera suggest that future predictive models should consider the influence of temperature on the relationship between sales and incidence.

Combinations of different products, with changes based upon season or other exogenous factors, may allow for enhanced disease-specific surveillance. In other areas with high diarrhea burden and poor health surveillance systems, like much of Sub-Saharan Africa, ORS use is low, particularly when care is provided by the private sector like the pharmacies and drug-sellers within this study [33,34]. Expanding this concept to populations beyond Bangladesh will require careful study of their care seeking behaviors and may require tracking multiple products simultaneously.

This study illustrates the potential to use first-line health providers in both epidemiologic surveillance and research within low-resource settings. While, significant hurdles remain to translate these results into practical and scalable surveillance tools, results from this study point toward many possible solutions.

## Supporting Information

S1 Fig Space-time cluster of cases (Panel A) and ORS sales within and outside of the cluster (Panel B). Panel A illustrates the two space-time clusters detected with the SatScan Space-Time permutation scan statistics (purple circles) along with the locations of 53 confirmed cases and the pharmacies where ORS sales were tracked. Panel B shows the individual pharmacy normalized ORS sales with pharmacies in Cluster #2 in purple and pharmacies outside of Cluster #2 in green. The thicker lines represent the loess-smoothed version of the pharmacy sales within and outside the cluster.
(TIF)

S2 Fig Geographic distance by sales trajectory similarity. Dots represent the pairwise sales-geographic distance between pharmacies and lines represent model predictions based on a generalized additive model (purple) and a linear spline model (orange) with a knot at 100m (knot location chosen based on GAM fit).
(EPS)

S3 Fig Frequency of reporting to the phone system by calendar week. Each plot shows the numbers of call reports by each pharmacy by week with a linear regression line fit through each. A linear random-effects model (random intercept for each pharmacy using lme4 package in R [35]) was also explored to understand whether there was a general downward trend in reporting frequency by week but we found no significant results.
(EPS)

S4 Fig Comparison of cumulative number of ORS customers as recorded by the paper- (green) and phone-based (red) ORS reporting systems. (EPS)

S5 Fig Relationship between community-wide excess ORS sales and mean daily temperature (a measured at Shahjalal International Airport). Dots represent data and line represents linear regression and 95% confidence intervals. In this model a one degree (C°) change in mean daily temperature is associated with a 0.06 unit increase in community-wide excess ORS sales (95% CI 0.047–0.080).
(EPS)

S1 Table Difference in AIC from unadjusted Poisson models with different lags compared to the best model. The first column represents models with confirmed cholera as an outcome, and the second column represents models with all diarrhea as an outcome. (Note: ΔAIC = AIC—AIC_min_)
(DOCX)

S2 Table Difference in AIC from adjusted Poisson models with different lags compared to the best model. The first column represents models with confirmed cholera as an outcome, and the second column represents models with all diarrhea as an outcome. (Note: ΔAIC = AIC—AIC_min_)
(DOCX)
