# Supplementary figures and images for: Tracking Cholera through Surveillance of Oral Rehydration Solution Sales at Pharmacies: Insights from Urban Bangladesh
Source: PLoS Negl Trop Dis. 2015 Dec 7;9(12):e0004230. doi: 10.1371/journal.pntd.0004230 (PMC4671575; doi:10.1371/journal.pntd.0004230)

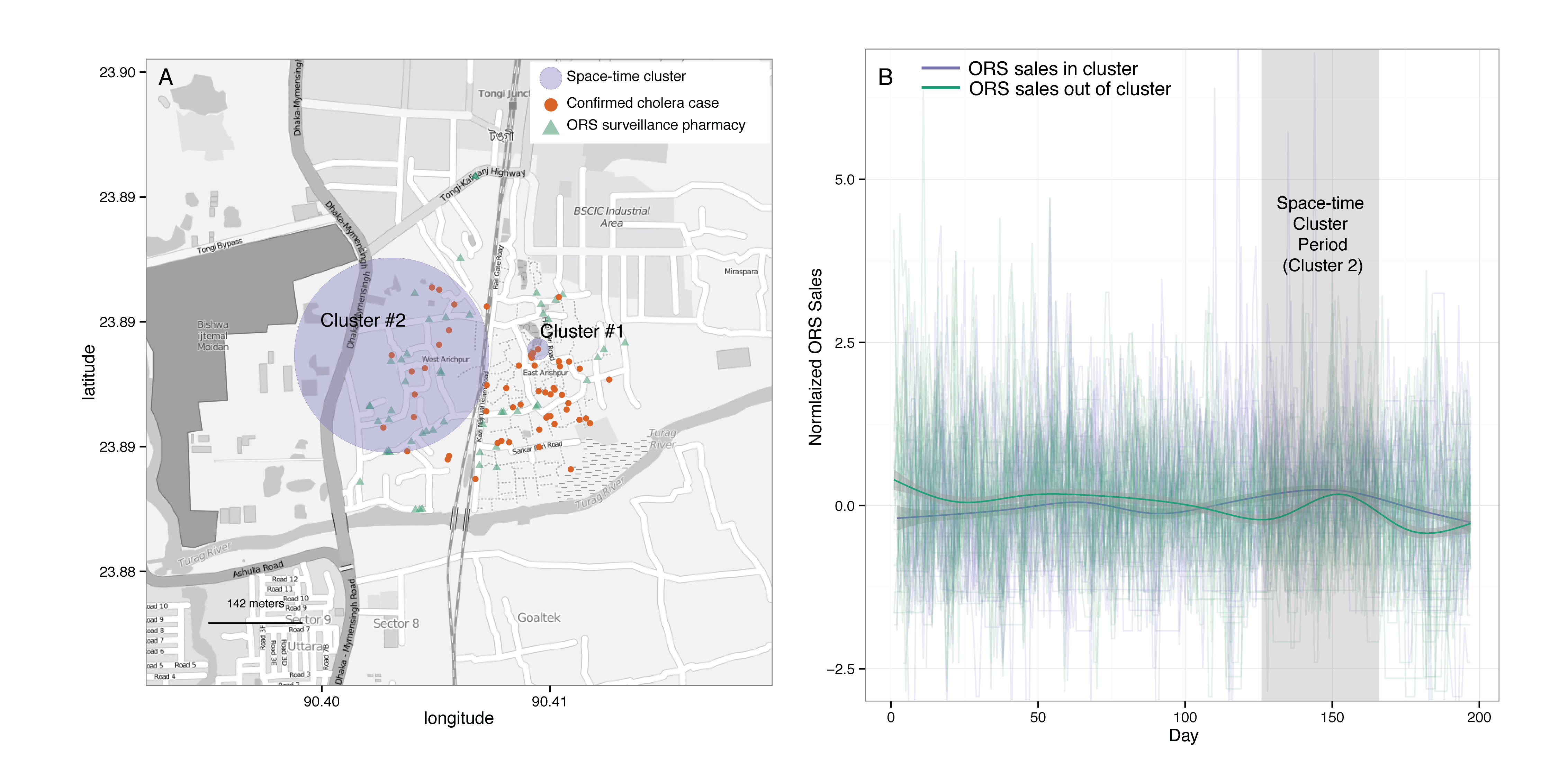

Supplement: S1 Fig — Panel A illustrates the two space-time clusters detected with the SatScan Space-Time permutation scan statistics (purple circles) along with the locations of 53 confirmed cases and the pharmacies where ORS sales were tracked. Panel B shows the individual pharmacy normalized ORS sales with pharmacies in Cluster #2 in purple and pharmacies outside of Cluster #2 in green. The thicker lines represent the loess-smoothed version of the pharmacy sales within and outside the cluster. (TIF) [file pntd.0004230.s001.tif]
