# Supplementary material for: Tracking Cholera through Surveillance of Oral Rehydration Solution Sales at Pharmacies: Insights from Urban Bangladesh
Source: PLoS Negl Trop Dis. 2015 Dec 7;9(12):e0004230. doi: 10.1371/journal.pntd.0004230 (PMC4671575; doi:10.1371/journal.pntd.0004230)
Supplement: S1 Table — The first column represents models with confirmed cholera as an outcome, and the second column represents models with all diarrhea as an outcome. (Note: ΔAIC = AIC—AICmin) (DOCX) [file pntd.0004230.s006.docx]

| Lag  (days) | ∆AIC  cholera | ∆AIC  all diarrhea |
| --- | --- | --- |
| 0 | 3.3 | 25.7 |
| 1 | 0.0 | 24.7 |
| 2 | 9.9 | 11.9 |
| 3 | 8.8 | 13.5 |
| 4 | 6.8 | 2.8 |
| 5 | 9.4 | 11.6 |
| 6 | 9.5 | 6.0 |
| 7 | 6.9 | 0.0 |
| 8 | 5.2 | 9.9 |
| 9 | 5.5 | 11.0 |
| 10 | 6.1 | 8.3 |
