# Supplementary material for: Tracking Cholera through Surveillance of Oral Rehydration Solution Sales at Pharmacies: Insights from Urban Bangladesh
Source: PLoS Negl Trop Dis. 2015 Dec 7;9(12):e0004230. doi: 10.1371/journal.pntd.0004230 (PMC4671575; doi:10.1371/journal.pntd.0004230)
Supplement: S2 Table — The first column represents models with confirmed cholera as an outcome, and the second column represents models with all diarrhea as an outcome. (Note: ΔAIC = AIC—AICmin) (DOCX) [file pntd.0004230.s007.docx]

| Lag  (days) | ∆AIC  cholera | ∆AIC  all diarrhea |
| --- | --- | --- |
| 0 | 1.6 | 20.6 |
| 1 | 0.0 | 18.2 |
| 2 | 6.6 | 9.6 |
| 3 | 5.9 | 9.5 |
| 4 | 5.2 | 4.6 |
| 5 | 6.5 | 8.6 |
| 6 | 6.6 | 5.4 |
| 7 | 4.5 | 0.0 |
| 8 | 3.7 | 5.6 |
| 9 | 5.5 | 11.0 |
| 10 | 6.1 | 8.3 |
